# Hyperuricemia in Children and Adolescents: Present Knowledge and Future Directions

**DOI:** 10.1155/2019/3480718

**Published:** 2019-05-02

**Authors:** Masaru Kubota

**Affiliations:** Department of Agriculture, Ryukoku University, Ohtsu, Shiga, Japan

## Abstract

Recent evidence suggests that hyperuricemia is an important condition in children and adolescents, particularly in association with noncommunicable diseases. This review aims to summarize our current understanding of this condition in pediatric patients. An analysis of serum uric acid reference values in a healthy population indicates that they increase gradually with age until adolescence, with differences between the sexes arising at about 12 years of age. This information should be taken into consideration when defining hyperuricemia in studies. Gout is extremely rare in children and adolescents, and most patients with gout have an underlying disease. The major causes of hyperuricemia are chronic conditions, including Down syndrome, metabolic or genetic disease, and congenital heart disease, and acute conditions, including gastroenteritis, bronchial asthma (hypoxia), malignant disorders, and drug side effects. The mechanisms underlying the associations between these diseases and hyperuricemia are discussed, together with recent genetic information. Obesity is a major cause of hyperuricemia in otherwise healthy children and adolescents. Obesity is often accompanied by metabolic syndrome; hyperuricemia in obese children and adolescents is associated with the components of metabolic syndrome and noncommunicable diseases, including hypertension, insulin resistance, dyslipidemia, and chronic kidney disease. Finally, strategies for the treatment of hyperuricemia, including lifestyle intervention and drug administration, are presented.

## 1. Introduction

Hyperuricemia is a laboratory abnormality often observed in children and adolescents. However, because of the low diagnostic value of serum uric acid (hereafter described as “uric acid”) alone, uric acid levels may not be adequately considered by pediatricians. More attention has been drawn to hyperuricemia in children and adolescents by several recent studies reporting its association with obesity and noncommunicable diseases (NCDs), especially cardiovascular disorders. A search of the literature found only two reviews of hyperuricemia in children and adolescents [1, 2]. Although these reviews are comprehensive and well summarized, they have two major drawbacks. First, they do not adequately address the associations between hyperuricemia and NCDs. Second, recent findings of genetic studies on hyperuricemia are not fully discussed. Therefore, this review aims to cover these topics and proposes future directions for research on hyperuricemia in children and adolescents.

## 2. Database

Articles published from 2000 to 2018 were retrieved in searches of Medline and Web of Science. The search methodology consisted of both controlled vocabulary as in the National Library of Medicine MeSH and the keywords uric acid/hyperuricemia and child/adolescent. Reviews and case reports published before 2000 are included if considered to be of particular importance.

## 3. Reference Values of Uric Acid in Children and Adolescents

In adults, serum uric acid >7.0 mg/dL is widely used as the definition of hyperuricemia, considering the solubility of uric acid [3, 4]. However, uric acid levels in children and adolescents change during development. Therefore, age- and sex-related reference values for uric acid should be considered for defining hyperuricemia in children and adolescents. Summarizing previous reports, the following developmental changes in uric acid levels have been identified although the absolute uric acid values differ marginally from report to report [1, 5–7]. Uric acid levels increase gradually from birth to the end of elementary school age. Subsequently, levels rise sharply in males and slightly in females, creating a significant difference between the sexes. For reference, data from two studies [1, 6] are shown in Table 1.

## 4. Conditions Causing Hyperuricemia

### 4.1. Gout

Large-scale epidemiological studies of gout in children and adolescents are quite limited. In a study using data in the UK General Practice Research Database (1990–1999), Mikuls et al. report that the incidence of gout in individuals <25 years was 12 in 255,950 men and 1 in 246,346 women [8]. The incidence between 2007 and 2015 in Korea was 2 to 3 in 100,000 persons aged 0–9 years and 9 to 20 in 100,000 persons aged 10–19 years [9]. Our nationwide questionnaire survey of pediatric departments identified only seven cases of gout in more than 2,300,000 inpatients and outpatients under 15 years of age [10]. All patients with gout in our study were found to have at least one underlying disorder. Previous case reports have shown that gout in children or adolescents was associated with comorbidities such as Down syndrome [11], glycogen storage disease [12], renal transplantation [13], leukemia [14], and methyl malonic acidemia [15] (Table 2).

### 4.2. Chronic Disease

#### 4.2.1. Metabolic Diseases

Abnormalities in the metabolism of purine or related compounds have been shown to cause hyperuricemia in infancy or childhood [16, 17]. These abnormalities include complete hypoxanthine-guanine phosphoribosyl transferase (HGPRT) deficiency (Lesch–Nyhan syndrome), partial HGPRT deficiency (Kelly–Seegmiller syndrome), adenine phosphoribosyl transferase (APRT) deficiency, phosphoribosyl pyrophosphate (PRPP) synthetase overactivity, and myoadenylate deaminase deficiency. The most common cause of hyperuricemia is the overproduction of uric acid [1, 17]. Some patients with Lesch–Nyhan syndrome, Kelly–Seegmiller syndrome, or PRPP synthetase overactivity exhibit gout in early childhood or adolescence [1]. Glycogen storage diseases, especially type I, III, V, and VII, also cause hyperuricemia. Muscle exertion in these diseases is thought to result in accelerated degradation of muscle purine nucleotides [1].

#### 4.2.2. Down Syndrome

Down syndrome, seen in approximately one case per 800–1000 live births, is the most common chromosomal abnormality. The association between Down syndrome and hyperuricemia was described quite early [18]. Since then, several reports have indicated the higher prevalence of hyperuricemia in patients with Down syndrome [19, 20]. Particularly, Kashima et al. report that hyperuricemia in Down syndrome appears in early infancy, according to their age-specific reference values [20]. The underlying cause of this association is proposed to be elevated levels of purine metabolizing enzymes [21] and lifestyle related factors such as obesity or low physical activity [20]. Recently, Garlet et al. presented the hypothesis that the elevated uric acid level is a compensatory response to the redox imbalance accompanying Down syndrome [22].

#### 4.2.3. Congenital Heart Diseases

Congenital heart diseases, especially cyanotic heart diseases, are often associated with hyperuricemia [6, 23]. Polycythemia, chronic hypoxia, and the resultant increased purine catabolism are proposed to be the primary pathogenetic mechanisms underlying this association [1]. Rodríguez-Hemández et al. recently postulated that BMI, impaired renal function, cyanosis, and the use of diuretics are risk factors for hyperuricemia in patients with congenital heart diseases [24].

#### 4.2.4. Genetic Disorders

Familial juvenile hyperuricemic nephropathy (FJHN) is characterized by juvenile onset of hyperuricemia, gout, and progressive nephropathy [25]. Hart et al. first determined that a mutation in the uromodulin gene is responsible for FJHN [26]. A genome-wide association study revealed that single-nucleotide polymorphisms in the uric acid transporter genes (ABCG2 and SLC2A9) cause hyperuricemia and gout by altering urinary uric acid clearance [27]. Apolipoprotein E gene polymorphisms are also associated with primary hyperuricemia, as identified by Wu et al. in a Chinese pediatric population of 770 subjects [28].

### 4.3. Acute Diseases

#### 4.3.1. Gastroenteritis

Our investigation of conditions associated with hyperuricemia in more than 9000 pediatric inpatients revealed that gastroenteritis is the most common [6]. Of the agents causing gastroenteritis, rotavirus infection is most likely to cause hyperuricemia [29, 30]. Dehydration is the suspected cause of hyperuricemia in gastroenteritis; uric acid levels return to normal levels readily after hydration treatment, without the use of antihyperuricemia drugs. In addition, Matsuo et al. suggest an additional possible mechanism involving decreased intestinal uric acid excretion via ABCG2, caused by gastroenteritis-induced injury of the intestinal epithelium [31].

#### 4.3.2. Bronchial Asthma (Hypoxia)

Bronchial asthma, especially during an acute attack, is a common cause of hyperuricemia in pediatric patients [6]. Abdulnaby et al. demonstrated that the degree of serum uric acid elevation may serve as a useful marker of the severity of bronchial asthma [32]. Hypoxia and dehydration are considered possible underlying causes of hyperuricemia in bronchial asthma. In a study of sleep-disordered breathing in obese children and adolescents, the degree of arterial oxygen saturation was found to correlate with uric acid levels [33].

#### 4.3.3. Malignant and Hematological Disorders

Pediatric patients with malignant disorders, particularly hematological diseases such as leukemia and lymphoma, are at risk for hyperuricemia [34, 35]. This risk may be due in part to the large tumor burden at onset characteristics of pediatric malignancies [35]. In addition, the higher susceptibility of children to chemotherapeutic agents places them at greater risk than adult patients for tumor lysis syndrome (TLS) [36].

Hemolytic anemia, such as hemolytic uremic syndrome [37] and sickle cell anemia [38], causes hyperuricemia. Erythrocyte overdestruction and hyperlacticacidemia may play a role in the occurrence of hyperuricemia. Notably, uric acid levels sometimes exceeded 20 mg/dL, necessitating peritoneal dialysis in patients with hemolytic uremic syndrome [37].

#### 4.3.4. Side Effects of Drugs

Several drugs such as diuretics (thiazide), anticonvulsants (valproate and phenobarbital), cyclosporine, theophylline, and pyrazinamide have been reported to increase uric acid levels in children and adolescents [1, 13, 39–41]. Although the underlying causes are not fully understood, renal tubular reabsorption of uric acid, dehydration, and increased purine catabolism has been postulated [1, 42].

### 4.4. Lifestyle-Related Disorders

#### 4.4.1. Obesity

Obesity in children and adolescents is the global problem seen in both developed and developing countries [43, 44]. Results of large-scale studies on the prevalence of hyperuricemia in a general population of children and adolescents are summarized in Table 3 [45–49]. Generally, the prevalence of hyperuricemia tends to be higher in Western countries than in Asian countries and in males than in females. The limited reports in an obese population demonstrated a prevalence of several folds higher that in a general population; however, these reports had drawbacks, including smaller cohorts of obese than general populations and heterogeneity in the definition of obesity [50–53]. Furthermore, the mean uric acid levels turn out to be higher in an obese population [54, 55]. During over a 6-year follow-up period in children aged 6–12 years at study commencement, Kuwahara et al. showed that an excessive increase in BMI during that time was associated with significant uric acid elevation by early adolescence [56].

#### 4.4.2. Metabolic Syndrome (MS)

The association of hyperuricemia with MS is well documented in adults [57]. In a recent review of MS in children and adolescents, Bussler et al. demonstrated a prevalence of MS ranging from 6 to 39%, depending on the applied definition criteria [58]. They also identified hyperuricemia as an important comorbidity, together with nonalcoholic fatty liver disease and sleep restriction. In a cohort of 1370 adolescents aged 12–17 years divided into four quartiles based on the uric acid level, Ford et al. observed a sequentially higher prevalence of MS from the lowest quartile (<1%) to the highest quartile (21.1%) [45]. In 2284 Taiwanese children aged 6–12 years, uric acid levels were found to be a significant predictor of MS, exhibiting a 54% increased risk for MS for every 1 mg/dL increase in uric acid concentration [46]. The positive association of uric acid levels or hyperuricemia with the occurrence of MS has been reported in multiple countries, including Japan [50], Spain [51], and Brazil [52]. In addition, several studies report that the relationship between uric acid levels and MS in adolescents is dependent upon racial/ethnic and sex differences [59, 60].

### 4.5. Components of MS or NCDs

#### 4.5.1. Hypertension

Hypertension in relation to hyperuricemia has been most extensively investigated among components of MS or NCDs. In a cohort of 125 children aged 6–18 years being evaluated for hypertension, Feig and Johnson observed that uric acid levels correlate directly with blood pressure in untreated children and that a concentration >5.5 mg/dL in adolescents strongly suggest primary hypertension [61]. Numerous studies in different countries have subsequently confirmed this association between hyperuricemia and hypertension in children and adolescents [49, 55, 62, 63]. In the tracking of 449 children aged 3–7, Park et al. found that children with high uric acid levels at both 3 and 5 years had the highest systolic blood pressure at 7 years [64]. In addition, by the average follow-up period of 12 years, elevated childhood uric acid levels are shown to be associated with increased blood pressure in beginning in children and higher blood pressure levels that persist into adulthood [65]. Taking these reports into consideration, Yanik and Feig suggest the use of the uric acid level as a possible biomarker for diagnosing essential hypertension in children [66].

#### 4.5.2. Insulin Resistance

In an earlier study of Japanese obese children aged 7–15 years, fasting insulin levels correlated positively with uric acid levels [67]. After adjustment by age, sex, and BMI, uric acid levels were found to be positively associated with insulin resistance as evaluated by homeostasis model assessment (HOMA-R) and negatively associated with adiponectin concentration [54]. A study of Greek obese youths indicates an association between hyperuricemia and HOMA-R [68].

#### 4.5.3. Dyslipidemia and Atherosclerosis

Denzer et al. observed that triglycerides and the cholesterol/HDL ratio correlated positively with uric acid levels in a cohort of 269 obese children whose BMI was >90^th^ percentile [69]. In a study of different ethnic groups in Taiwan, hypertriglycemia and hypercholesterolemia were associated with hyperuricemia, but the tendency differed between ethnic groups (Aborigines vs. non-Aborigines) [70]. Hyperlipidemia is associated with an increased risk of atherosclerosis in children and adolescents [71]. Pacifico et al. showed that the carotid intima-medial thickness, an indicator of atherosclerosis, was elevated in participants in the fourth quartile of uric acid compared to those in the first, second, and third quartiles [72]. Furthermore, in Japanese obese children, uric acid levels were shown to correlate positively with lipids and negatively with flow-mediated dilatation of the brachial artery [73]. Together, these results suggest that hyperuricemia in obese children may be a marker for early atherosclerosis.

#### 4.5.4. Chronic Kidney Disease (CKD)

A recent review suggests that uric acid plays a role in the pathogenesis of CKD in children [74]. Rodenbach et al. demonstrated that hyperuricemia was an independent risk factor for faster progression of CKD in a cohort of over 600 children and adolescents over a 5-year period [75]. Furthermore, lowering of uric acid levels with allopurinol over a 4-month period improved eGFR independently in children with stage 1–3 CKD [76].

## 5. Treatment of Hyperuricemia

Apart from a causal relationship, it is clear that hyperuricemia and those four components of NCDs described above are closely associated each other. Hyperuricemia in children and adolescents is a target of treatment, considering the report demonstrating that pediatric patients with hyperuricemia are at increased risk of mortality, particularly due to kidney and cardiovascular diseases [77].

### 5.1. Lifestyle Intervention

Since obesity is a major cause of hyperuricemia in otherwise healthy children and adolescents, programs for reducing body weight by lifestyle intervention (dietary, physical activity, and behavioral changes) are important [78]. To my best knowledge, however, only two studies addressing this issue are present in the literature. Togashi et al. demonstrated that uric acid levels decreased significantly in 33 obese children after diet plus exercise treatment for 3 months [79]. Furthermore, a one-year weight reduction program in a cohort of 10–17-year-olds was reported to decrease uric levels in 86% of the females and 67% of the males [80]. Although the effect of programs of intervening a lifestyle on obesity is promising, the effect on hyperuricemia requires further investigation over a longer observation period.

### 5.2. Xanthine Oxidase Inhibitors

Allopurinol, an inhibitor of xanthine oxidase, is an old drug commonly used to treat a variety of pediatric diseases [17, 25], including HGPRT deficiency [81], APRT deficiency [82], glycogen storage disease type Ia [83], and FJHN [84]. In addition, the efficacy of allopurinol treatment alone [85] or in combination with enalapril [86] in reducing blood pressure has been investigated in children with hyperuricemic hypertension. However, allopurinol should be used with caution as it can cause severe skin side effects, including Stevens–Johnson syndrome (SJS). The association of allopurinol-induced SJS with human leukocyte antigen (HLA)-B ∗ 5801 has been identified [87].

Febuxostat is a newly developed nonpurine, selective inhibitor of xanthine oxidase [88]. Kaku and Nishimura administered febuxostat to 16 children with CKD and observed a renal protective effect accompanied by the lowering of uric acid levels [89]. When used as prophylaxis for TLS in pediatric hematological malignancies, an effect comparable to that of allopurinol was observed [90]. Further investigation is required to determine the efficacy and safety of febuxostat for use in children and adolescents.

### 5.3. Uric Acid Oxidase (Rasburicase)

A recombinant uric acid oxidase, rasburicase, is widely used for the prevention of hyperuricemia observed in TLS at diagnosis or during treatment of a variety of malignancies in children [91, 92]. Rasburicase treatment achieved a greater and more rapid decline in uric acid levels than did allopurinol [91]. Despite the efficacy of rasburicase in malignancy-induced TLS, Cheuk et al. raised concerns about its serious side effects, including hypersensitivity and hemolysis [93].

## 6. Conclusion and Future Directions

This review raises three points that should be considered to improve and shape the direction of future research on hyperuricemia in children and adolescents. First, the reference values of uric acid in children and adolescents change with age, with a difference between the sexes arising at about 12 years of age. Therefore, the definition of hyperuricemia used in data analysis should take these factors into account. Second, future studies should address the question of how hyperuricemia arising in childhood or adolescence affects health in adulthood, especially regarding NCDs. Large cohort, long-term follow-up studies are needed to answer this question. Third, the treatment of hyperuricemia in children and adolescents should be investigated with the aim of standardization, including recommendations as to when uric acid-lowering treatment should be initiated and which drugs are most suitable. These factors are particularly important with respect to chronic diseases that cause hyperuricemia starting early in childhood. The efficacy of treatment through lifestyle intervention also should be investigated. As our understanding of the importance of hyperuricemia in childhood improves, pediatricians should pay greater attention to hyperuricemia in the clinical setting.

## Figures and Tables

**Table 1:** Reference values of uric acid in children and adolescents.

	Uric acid (mg/dL)			
	Sex^*∗∗*^	Age (years)	Mean	SD
Wilcox [1]^*∗*^		<5	3.6	0.9
		5∼10	4.1	1.0
	Male	12	4.4	1.1
		15	5.6	1.1
		18	6.2	0.8
	Female	12	4.5	0.9
		15	4.5	0.9
		18	4.0	0.7

Kubota [6]		<1	2.9	0.9
		1∼3	3.3	0.8
		4∼6	3.6	1.0
		7∼9	4.2	0.9
	Male	10∼12	4.5	0.9
		>13	5.6	1.0
	Female	10∼12	4.1	0.8
		>13	4.3	0.9

^*∗*^The number within brackets indicate the reference number. ^*∗∗*^The blanks in this column indicate both sexes.

**Table 2:** Diseases/disorders causing hyperuricemia in children and adolescents.

1. Gout
2. Chronic diseases
(a) Metabolic disease
Hypoxanthine-guanine phosphoribosyl transferase (HGPRT) deficiency
(complete, Lesch–Nyhan syndrome; partial, Kelly–Seegmiller syndrome)
Adenine phosphoribosyl transferase (APRT) deficiency
Phosphoribosylpyrophosphate (PRPP) synthetase overactivity
Myoadenylate deaminase deficiency
Glycogen storage diseases (types I, III, V, and VII)
Acyl-coenzyme A dehydrogenase deficiency
(b) Down syndrome
(c) Congenital heart disease (especially cyanotic diseases)
(d) Genetic diseases
Familial juvenile hyperuricemic nephropathy (FJHN)
3. Acute diseases
(a) Gastroenteritis (especially Rotavirus infection)
(b) Bronchial asthma (especially on attacks)
(c) Malignant disorders (tumor lysis syndrome)
(d) Hemolytic anemia
(e) Drugs
Diuretics (thiazide)
Theophylline
Anticonvulsants (valproate and phenobarbital)
Cyclosporine
Pyrazinamide
4. Lifestyle-related disorders
(a) Obesity
(b) Metabolic syndrome

**Table 3:** Prevalence of hyperuricemia in a general or obese children and adolescents.

Author	Study year	Number	Age (years)	Sex	Uric acid (mg/dL)	Hyperuricemia (%)
*General population*						
Ford et al. [45]^*∗*^	1999–2002	1370	12∼17	Both	≧5.5	30.2
					≧6.0	22.2
					>7.0	6.5
Lee et al. [46]	2001–2002	2284	6∼12	Male	≧7.0	26.5
				Female		18.8
Shatat et al. [47]	2005–2008	1912	13∼18	Both	>6.0	19.3
Kawasaki et al. [48]	2011–2012	29714	<15	Male	≧7.1	5.4
					≧8.0	1.6
				Female	≧7.1	0.45
					≧8.0	0.15
Li et al. [49]	2015	4073	3∼6	Male	≧5.1	11.8
				Female		8.3

*Obese population*						
Tang et al. [50]	2005–2008	1027	6∼14	Male	≧5.9∼7.0	24.4
				Female	≧5.9∼6.2	15.2
Modino et al. [51]	Not shown	148	5∼19	Both	≧5.5	53
Cardoso et al. [52]	2009–2010	129	<18	Both	>5.5	12.4
Ságodi et al. [53]	Not shown	162	10∼14	Both	Unknown	38.3

^*∗*^The number within brackets indicate the reference number.
